# Design of Surface Plasmon Resonance-Based D-Type Double Open-Loop Channels PCF for Temperature Sensing

**DOI:** 10.3390/s23177569

**Published:** 2023-08-31

**Authors:** Shuangyan Gao, Kaihua Wei, Hua Yang, Yongjian Tang, Zao Yi, Chaojun Tang, Bin Tang, Yougen Yi, Pinghui Wu

**Affiliations:** 1 Joint Laboratory for Extreme Conditions Matter Properties, Southwest University of Science and Technology, Mianyang 621010, China; 18838966730@163.com (S.G.); tangyongjian2000@sina.com (Y.T.); 2 School of Automation, Hangzhou Dianzi University, Hangzhou 310018, China; weikaihua@hdu.edu.cn; 3 School of Science, Lanzhou University of Technology, Lanzhou 730050, China; hyang@lut.edu.cn; 4 School of Chemistry and Chemical Engineering, Jishou University, Jishou 416000, China; 5 College of Science, Zhejiang University of Technology, Hangzhou 310023, China; chaojuntang@zjut.edu.cn; 6 School of Microelectronics and Control Engineering, Changzhou University, Changzhou 213164, China; btang@cczu.edu.cn; 7 College of Physics and Electronics, Central South University, Changsha 410083, China; yougenyi@csu.edu.cn; 8 Key Laboratory of Information Functional Material for Fujian Higher Education, Quanzhou Normal University, Quanzhou 362000, China

**Keywords:** Core-Metal-Analytes, double open-loop channels, surface plasmon resonance, temperature sensing

## Abstract

Here, we document a D-type double open-loop channel floor plasmon resonance (SPR) photonic crystal fiber (PCF) for temperature sensing. The grooves are designed on the polished surfaces of the pinnacle and backside of the PCF and covered with a gold (Au) film, and stomata are distributed around the PCF core in a progressive, periodic arrangement. Two air holes between the Au membrane and the PCF core are designed to shape a leakage window, which no longer solely averts the outward diffusion of Y-polarized (Y-POL) core mode energy, but also sets off its coupling with the Au movie from the leakage window. This SPR-PCF sensor uses the temperature-sensitive property of Polydimethylsiloxane (PDMS) to reap the motive of temperature sensing. Our lookup effects point out that these SPR-PCF sensors have a temperature sensitivity of up to 3757 pm/°C when the temperature varies from 5 °C to 45 °C. In addition, the maximum refractive index sensitivity (RIS) of the SPR-PCF sensor is as excessive as 4847 nm/RIU. These proposed SPR-PCF temperature sensors have an easy nanostructure and proper sensing performance, which now not solely improve the overall sensing performance of small-diameter fiber optic temperature sensors, but also have vast application prospects in geo-logical exploration, biological monitoring, and meteorological prediction due to their remarkable RIS and exclusive nanostructure.

## 1. Introduction

Temperature is a fundamental parameter in many fields, such as resource development, climate change, and medical diagnosis [1,2]. Temperature sensors can be divided into two main patterns: electrical transducer and photosensors.

Electrical sensors have been widely researched and applied as early as in practice, but there are still some disadvantages, such as the need for extra systems, large bulk, high costs, high power waste, weak multiplexing, remote monitoring capabilities, etc. [3]. In addition to these reasons, they are also more susceptible to the uncertainty of the environment itself, which not only adds an extra burden to the sensing device, but also affects the sensing sensitivity. An electrical transducer can also come in for electromagnetic (EMC) interference during signal transmission, leading to inaccurate sensing results [4,5,6].

As far as light sensors such as optical fiber sensors (OFS) are concerned, they have more outstanding benefits than the electrical sensors noted in the preceding paragraph. In particular, they have the characteristics of a small size and strong anti-interference, far flung sensing can be realized, and there is the possibility for prepared integration and reuse [7]. Its outstanding sensing characteristics allow it to be used in more sophisticated and hostile environments, for example, underwater and in environments sensitive to electric induction. With the development of photonic fiber technology [8,9], it has been found that combining photonic crystal fiber (PCF) with plasmon resonance (SPR) technology can overcome the shortcomings of traditional optical sensors, such as not being able to bend at large angles and at a short transmission distance. These excellent characteristics of SPR-PCF sensors can effectively improve sensing sensitivity and show high efficacy in many fields.

SPR is a touchy surface analysis technique, and when light is absolutely mirrored on the surface of the prism and metallic film, it will form a dying wave in the photophobic medium, and there is a positive plasma wave in the medium (assuming a metal medium). When the two waves resonate, the detected reflected mild intensity is considerably reduced. That is, the coupling generates a loss and achieves the purpose of sensing at the identical time [10,11,12]. In other words, SPR is the resonance phenomenon between electromagnetic waves (EW) generated by free electrons on the surface of metals and plasma waves on the surface. SPR technological know-how is widely used to allow the sensing of physical parameters due to the strongly excited floor plasmon polaron (SPP) mode with a robust electromagnetic discipline and sensitivity to small modifications in the analyte refractive index [13,14].

PCF is a new mannequin of optical fiber that transmits mild waves by using introducing periodically organized air holes in the fiber [15]. Different stomatal arrangements of PCF can result in it exhibiting different optical properties [16]. SPR coating with metal film, using the characteristics of PCF, is far better than conventional SPR sensors in terms of sensing performance [17]. In recent years, SPR-PCF pickup has been vastly used to take a look at many bodily parameters, such as the refractive index, temperature, magnetic field, etc. [18,19,20].

Currently, temperature-sensitive materials include oils [21], alcohols [22], and PDMS [23], among others. In contrast, PDMS has achieved excellent results in the field of temperature sensing due to its stability and linearity with temperature [23]. This paper is a study of temperature sensing with the help of PDMS.

In this paper, we propose a D-type double open-loop channel SPR-PCF temperature sensor to achieve a highly sensitive sensing of temperature by using PDMS. The sensor has two polished planes with an elliptical recess on each face, forming double open-loop channels. Two channels are coated with an Au film and the fibers are surrounded by polydimethylsiloxane (PDMS) as a temperature sensing medium. For better sensing, we designed polished surfaces and recesses. The sensor was then subjected to finite element analysis to investigate its modal characteristics, structural parameters, and sensing performance. Our research results indicate that the PCF sensor has good temperature sensitivity ($S_{\lambda }\;\geq \;3.$75 nm/°C) and RIS ($S_{\lambda }\;\geq \;4$800 nm/RIU). The sensitivity of the PCF sensor is higher than the present temperature sensor (such as the electromagnetic sensor, traditional light sensor, etc.), and because of its simple nanostructure, convenient manufacturing, high environmental applicability, high portability, etc., it not only has practical potential, but also meets the current demand for a high-sensitivity temperature sensor, and is an innovative development of the temperature sensor.

## 2. Nanostructure and Principle

**Figure 1:**
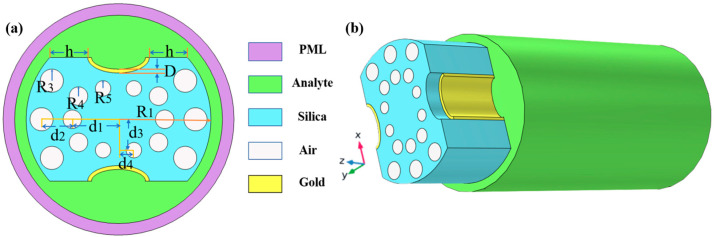
(**a**) The PCF sensor’s dimension and its geometrical parameters: d = 30 nm, R_1_ = 12 μm, R_3_ = 1.5 μm, R_4_ = 1.2 μm, R_5_ = 1 μm, h = 4√5 μm, d_1_ = 6 μm, d_2_ = 4 μm, d_3_ = 4 μm, d_4_ = 2 μm. (**b**) Stereogram of a D-type double open-loop channel PCF.

**Figure 2:**
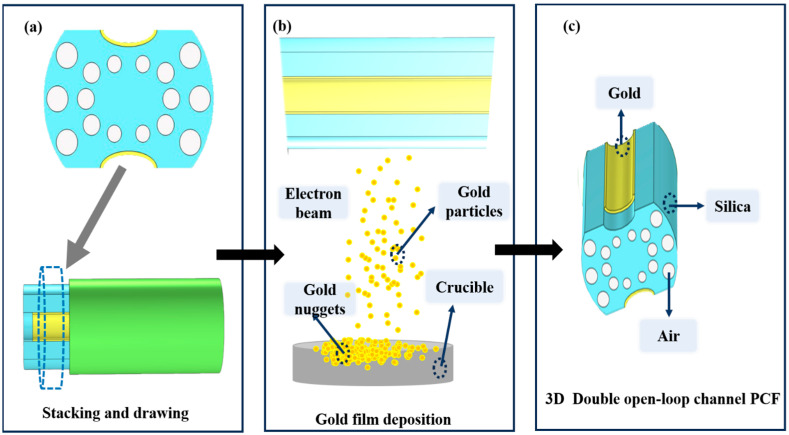
Schematic of D-type double open-loop channel PCF preparation. (**a**) is fiber cross-section, (**b**) is the ideal gold plating process, (**c**) is a D-type double open-loop channel PCF in 3D mode.

Figure 1 indicates a cross-section of the D-type dual-open-loop channel SPR-PCF designed in this paper. The PCF has a radius of 12 μm and can be processed with the aid of lofting and drawing. The grinding wheel polishing approach is used to graph grooves in the airplane of the top and bottom of the PCF (the aircraft is 8 μm from the middle of symmetry above and below) [24,25]. The Au film was coated on two open-loop channels by way of chemical vapor deposition or magnetron sputtering, and the magnetron sputtering technique used to be generally selected in the experiment [26,27,28]. In Figure 1, h represents the width of the surface of the non-grooved part after polishing (one side), and D represents the thickness of the Au film. The specific parameter settings are shown in Table 1. R_3_, R_4_, and R_5_ point out the radii of holes 1, 2, and 3, respectively, d1 varies from the hub of the fiber core for the No. 1 hole, d_2_ is the range from the hub of air holes 1 and 2, d_3_ is positioned faraway vertically towards the cable’s hub for the No. 3 hole, and d_4_ is the horizontal distance. The radii of the R_3_ pore (No. 1 hole) and R4 pore (No. 2 hole) are rotationally symmetrical distributions around the core; the center of rotation is the center of the core, and the rotation angle is 30°. Among them, the No. 3 hole is the most special hole, and its distribution as well as size, etc., have the most influence on the SPR pattern compared to other air holes. The ideal preparation process of the dual open-loop channel PCF proposed in this paper is shown in Figure 2.

During the analysis, the index of refraction of the air hole is put up, and Sellmier’s equation may be used to express the connection between the silica’s coefficient of refraction and the length of the spectrum [29]:

(1) $$n^{2}\left(\lambda \right)=1+\frac{B_{1}\lambda^{2}}{\lambda^{2}-C_{1}}+\frac{B_{2}\lambda^{2}}{\lambda^{2}-C_{2}}+\frac{B_{3}\lambda^{2}}{\lambda^{2}-C_{3}}$$

In Sellmier’s equation, *λ* is the wavelength. The constants *B*_1_, *B*_2_, *B*_3_, *C*_1_, *C*_2_, and *C*_3_ are 0.6961663, 0.407942, 0.8974794, 0.0684043 μm, 0.1162414 μm, and 9.896161 μm, respectively.

The relative dielectric constant of the Au layer is determined by the Drude-Lorentz model [30]:

(2) $$\varepsilon \left(\omega \right)=\varepsilon_{\infty }-\frac{\omega_{D}^{2}}{\omega \left(\omega +j\gamma_{D}\right)}+\frac{\Delta \varepsilon \cdot \Omega_{L}^{2}}{\left(\omega^{2}-\Omega_{L}^{2}\right)+j\Gamma_{L}\omega }$$

In particular, $\varepsilon_{\infty }$ = 5.9673 is the powerful dielectric constant and Δε = 1.09 is the balance element. The angular frequency is ω. $\omega_{D}$ = 4227.2π THz and $\gamma_{D}$= 31.84π THz represent the plasma frequency and reduction period, respectively. $\Omega_{L}$ = 1300.14π THz is the oscillator strength and $\Gamma_{L}$= 209.72π THz is the spectral width.

**Figure 3:**
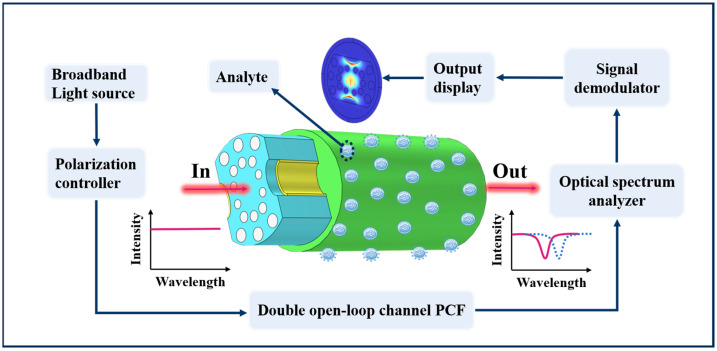
The working diagram of the sensor in this paper.

A gold film is used to excite SPR in this paper. The SPR effect is built on the basis of coupled-mode theory (CMT) [31]. When SPR occurs, coupling between modes is generated. It is possible to express the coupled mode theory as [32]:

(3) $$\left\{\begin{array}{c} \frac{\mathrm{d}E_{1}}{\mathrm{d}z}=i\beta_{1}E_{1}+i\kappa E_{2}\\ \frac{\mathrm{d}E_{2}}{\mathrm{d}z}=i\beta_{2}E_{2}+i\kappa E_{1} \end{array}\right.$$

where $E_{1}$ and $E_{2}$ are the electric fields’ strength of the heart mode and SPP mode. The propagation frequencies for the primary type and SPP mode are $\beta_{1}$ and $\beta_{2}$, correspondingly. z is the transmission distance and κ represents the interaction intensity. The schematic diagram of this paper is shown in Figure 3. When the phase matching conditional tense is contented, $\beta_{1}$ and $\beta_{2}$ are equal [33]. The electric fields of the heart and SPP modes follow the same trend, and when a lot of energy from the Y-POL mode is linked to the higher and lower order SPP modes, the loss of the Y-POL mode increases dramatically and the SPR peak appears in the spectrum [34]. Analysis of the SPR-PCF sensor’s sensing capabilities may be conducted using the degradation spectroscopy of the core mode. The put on and tear is associated with the core sample through the following equation [35]:

(4) aloss=8.686×2πλIm⁡(neff)×107dB/cm

where λ is the wavelength and Im⁡(neff) is the fictitious portion of the core mode’s ERI.

Theoretically, when the refractive index of PDMS changes, it purposes an alternate in the nice index of the core and SPP patterns. In addition, the change in the segment matching scenario leads to a shift in the SPR height [36,37,38]. The SPP mode is more sensitive to the exchange of the PDMS refractive index than the core mode. As a result, two notches and polished surfaces are designed in this paper, which now no longer solely deliver the Au film nearer to the core, but also promote each and every other, making top use of the sensitive traits of the SPP mode and improving the temperature sensitivity of the fiber sensor. The response of the PCF sensor can be evaluated by means of the skill of the volume of the exchange of the SPR resonant wavelength with the index of refraction of the measured object, which is expressed as Equation (5) [39]:

(5) $$S_{\lambda }=\frac{\Delta \lambda_{peak}}{\Delta n_{a}}\left(nm∕RIU\right)$$

where $\Delta \lambda_{peak}$ is the range of resonant wavelength deviation and $\Delta n_{a}$ is the refractive index change in the analyte.

PDMS is a polymer material that has a high thermos-optic coefficient and easy processing. Hence, PDMS is used in combination with PCF as a sensitive material for temperature detection. The expression that follows can be used to demonstrate the connection among its coefficient of the refractive index and temperature [40]:

(6) $$n\left(T\right)=n_{0}+k\times \left(T-To\right)$$

where $n_{0}$ is set as 1.4176, To = 20 °C is the initial temperature, and *k* = −0.00045/°C is the thermo-optic coefficient. In this paper, the effects of metal layer thickness, groove long axis length, and hole radius No. 3 on sensitivity are discussed when *n* = 1.40775, that is, when the refractive index of PDMS is at a temperature of 30 °C.

## 3. Analysis of Mode Characteristics

**Figure 4:**
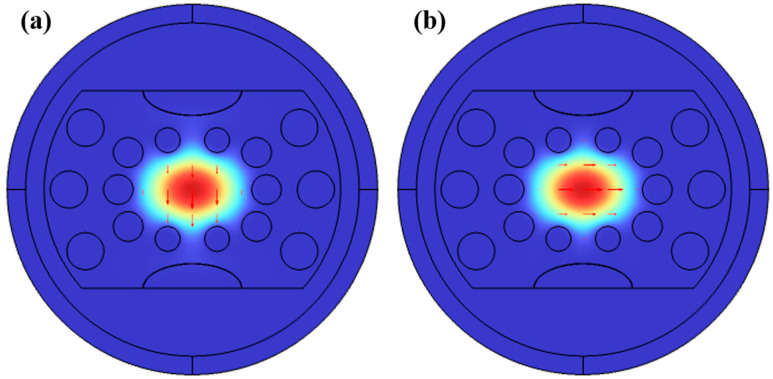
The electric field distributions for the Y-POL core mode (**a**) and the X-POL core mode (**b**). The red arrow is the direction of the electric field in this mode.

In this paper, numerical simulations of the D-type double open-loop channel PCF sensor nanostructure are performed using the control variables method with COMSOL 6.0 software based on finite element analysis [41,42]. Figure 4a,b exhibit the electric powered subject distribution of the Y-POL and X-POL imperative modes.

**Figure 5:**
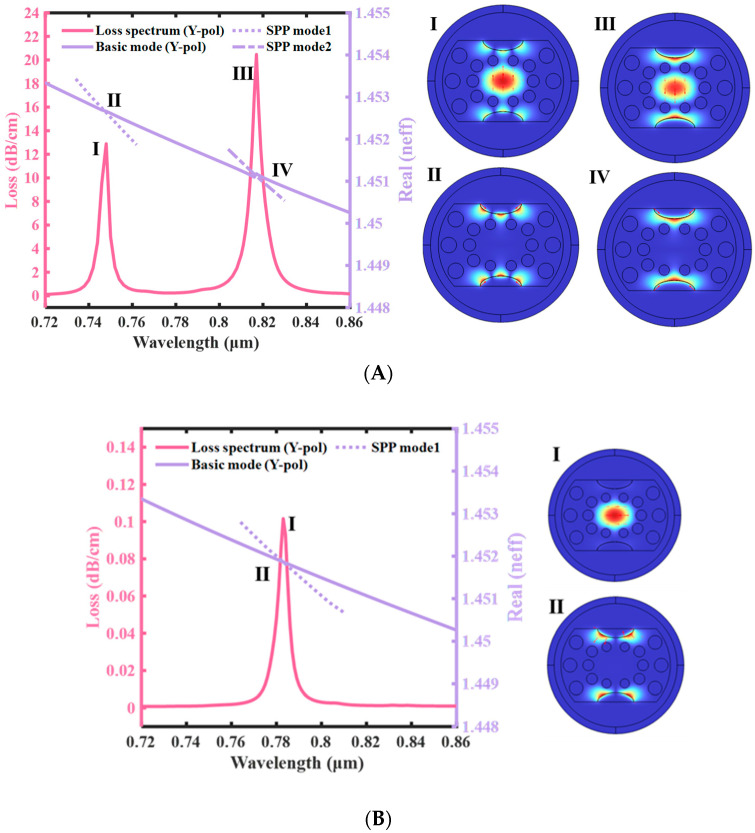
(**A**) *n* = 1.40775, D = 30 nm, aa = 4 μm, R_5_ = 1 μm. The ERI real part of the Y-POL core and SPP modes, and the loss spectra of the proposed D-type double open-loop channels PCF-SPR sensor. I and III of (**A**) are coupling modes of core mode and SPP mode, and II and IV are SPP modes. (**B**) *n* = 1.40775, D = 30 nm, aa = 4 μm, R_5_ = 1 μm. The ERI real part of the X-POL core and SPP modes, and the loss spectra of the proposed D-type double open-loop channels PCF-SPR sensor. I of (**B**) is coupling mode of core mode and SPP mode, and II is SPP mode.

This paper’s SPR-PCF sensor nanostructure is not highly symmetrical in terms of rotation, so the X-POL and Y-POL mode have one-of-a-kind positive refractive indices. The SPR top loss excited by using the two core modes is unique [43]. The Y-POL core mode in the PCF sensor nanostructure described in this study exhibits more distinct SPR issues and better sensing capabilities than the X-POL core mode (the metallic movie layer is in the Y direction, so the excited SPR height mode is extra obvious in the Y direction, as shown in Figure 5A,B). In addition, by solving Maxwell’s equations for steel dielectric surfaces, we observe that the SPP mode is a mode that is generally excited with the aid of the electric field, which is orthogonal to the metal layer’s outside [44,45,46]. The ERI of the Y-POL high-order SPP mode and low-order SPP mode (this paper normally studies Y-POL) at one-of-a-kind wavelengths, that is, the optical index’s real phase, which has an impact on the suggested sensor’s responsiveness and loss spectrum, is shown in Figure 4. The loss spectrum of the proposed sensor is shown in Figure 5, together with the real section of the ERI of the high-order SPP mode and low-order SPP mode of the X-POL at unusual frequencies. Comparing the two units of plots, we can observe that the loss top in X-POL is significantly less than the loss height in Y-POL by two orders of thousands. Moreover, in Figure 5, we can see that the lower-order SPR effect is more obvious than the higher-order one. After the above comparison, the main object of this paper is the low-order loss peak under Y-POL.

The PCF temperature sensor with double open-loop channels of D-type can be optimized with structural parameters to further improve the sensing performance.

## 4. Analysis of Nanostructure Parameters

After the above analysis, we found that the structural parameters of the PCF temperature sensor with a D-type double open-loop channel can be optimized to further improve the sensing performance [47]. It is worth noting that in the analysis process, the paper focuses on the Y-POL low-order SPP mode, considering the variety of situations that lead to the appearance of high-order loss peaks and the tendency to overlap phenomena that are not easy to find a pattern for.

### 4.1. Thickness of Au Film

**Figure 6:**
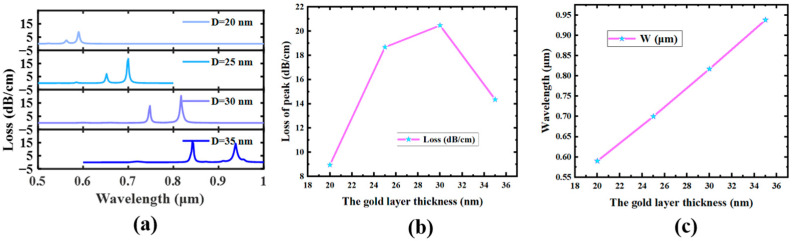
*n* = 1.40775, aa = 4 μm, R_5_ = 1 μm. (**a**) The effect of the change in elliptic semi-long axis aa on the loss peak. (**b**) The modification of the low-order mode’s highest point. (**c**) The change in wavelength where the maximum value of the loss peak of the low-order mode is located.

First of all, the iron movie layer’s thickness (the steel in this case is gold, the following are Au film) is the most important component affecting the peak loss, and we first discovered that it has an effect on law through the control variable method. We can see that in the range from 0.7 μm to 0.9 μm, the graphic demonstrates how varied Au movie thicknesses affect the loss spectrum. As proven in Figure 6, the resonant wavelength of SPR is red-shifted as the Au movie thickness in the fiber increases. We can additionally see that when the thickness of the Au movie is thin, the tremendous subject index of the Y-POL and SPP modes decreases, and the section matching point of SPR is blue-shifted. When D is between 20 and 30 nm, the ERI reduction of the SPP mode causes the SPR peak to move from 0.59 μm red to 0.817 μm, and the loss increases from 8.59 dB/cm to 20.47 dB/cm. This is shown in Figure 6. However, when D increases from 30 nm to 35 nm, the coupling of the Y-POL and SPP modes deviates from the most efficient coupling factor because the two evanescent fields are disturbed, and even disorderly loss peaks are generated [48]. Therefore, even if the SPR peak is red-shifted, the peak of the loss peak decreases instead. In order to ensure high sensitivity and high detection accuracy, D = 30 nm is chosen in this paper.

### 4.2. Radius of the Air Hole

**Figure 7:**
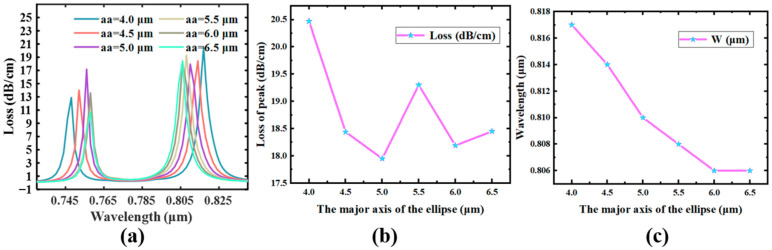
*n* = 1.40775, D = 30 nm, R_5_ = 1 μm. (**a**) The effect of the variation of elliptical long axis aa on the loss peak. (**b**) The modification of the low-order mode’s highest point. (**c**) The change in wavelength where the maximum value of the loss peak of the low-order mode is located.

Furthermore, we examined the ellipse’s semi-long axis. As the semi-major axis of the ellipse increases, although the low-order SPR loss top is blue-shifted, its peak variant appears to be extra difficult. In the range from 0.73 μm to 0.84 μm, the variation of its loss spectra with the half-length axis of the ellipse notch in the PCF sensor is displayed in Figure 7. As the elliptical semi-long axis increases, the overall ERI of the core mode of Y-POL appears to decrease, and the SPR peak is blue-shifted according to the phase-matching condition. At the same time, its low-order SPR loss peak first has a small fluctuation in the overall downward trend, which is due to changing the semi-major line within the ellipsoid outcomes in the alternate of the position of the Au film and the leakage window [49,50]. This will not only affect the ERI of the Y-POL, but also its coupling with the Au film. Therefore, Figure 7a shows that the SPR loss peaks of the lower order and the SPR loss peaks of the higher order are changed. Overall, the highest peak-to-peak value of low-order SPR loss is detected at half-length axis aa = 4 μm, so the sensor’s responsiveness and precision for detection approach a double-optimal condition.

### 4.3. The Long Axis of The Ellipse

**Figure 8:**
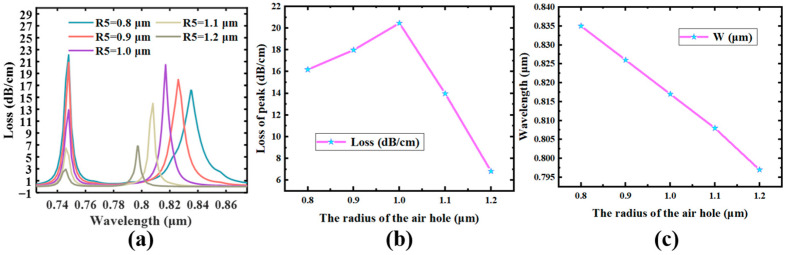
*n* = 1.40775, D = 30 nm, aa = 4 μm. (**a**) Effect of the change in the size of the radius of hole #3 on the loss peak. (**b**) The modification of the low-order mode’s highest point. (**c**) The change in wavelength where the maximum value of the loss peak of the low-order loss peak is located.

Last but not least, we also analyzed the radius of the No. 3 gap in the range from 0.725 μm to 0.875 μm, and Figure 8 shows that its loss spectrum increases with the radius of the bound hole in the PCF, one main to a make bigger and then a minimize in its ERI. The blue shift of the low-order SPR top is additionally derived from the phase-matching condition. The wastage increases from 16.19 dB/cm to 20.47 dB/cm when the binding hole radius increases from 0.8 μm to 1.0 μm. This is because as the binding hole radius increases, it makes the core energy concentrate in the middle of the two holes and coupled with the Au film, reduces the energy dissipation. However, when the radius of the holes increases from 1.0 μm to 1.2 μm, the loss decreases from 20.47 dB/cm to 13.98 dB/cm. This is because the leakage window between the two binding holes is too small as the radius of the binding hole increases [51]. This leads to a reduction in the ERI of the Y-POL, and it is difficult to allow the core energy to come out of the window and couple with the Au film. Therefore, the low-order SPR loss peak-to-peak value increases and then decreases, as in Figure 8b. After the above analysis, we chose to set the bound hole radius at 1.0 μm.

## 5. Temperature Sensing Performance

### 5.1. Temperature Sensing Performance

**Figure 9:**
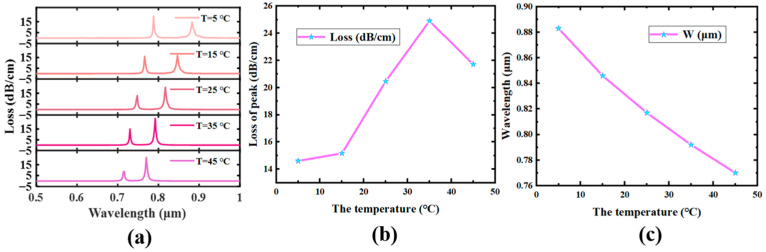
R_5_ = 1 μm, D = 30 nm, aa = 4 μm. (**a**) The effect of temperature change on the loss peak. (**b**) The change in the peak value of the low-order SPP mode. (**c**) The change in wavelength where the maximum fee of the loss top of the low-order SPP mode is placed.

Then, it explores the sensor capability across the temperature range from 5 °C to 45 °C. Due to the PDMS response temperature, Figure 9 suggests the variant of the SPR peak at different temperatures. The refractive index of PDMS drops with temperature, which also causes a fall in the ERI of its SPP mode, so the SPR peak is blue-shifted. PDMS is a high thermo-optical effect material, and its refractive index is very sensitive to temperature as it changes linearly with temperature, so the SPP mode’s propagation curve is the primary source of the substantial nonlinear exchange of the SPR peak. The low-order SPP mode’s sensitivity may be employed as the sensor’s temperature tolerance because, under the modification of the phase matching point, the SPP mode dispersion caused by thermal exchange causes the SPR peak to move to the blue [52,53]. Temperature changes lead to changes in the analyte’s refractive index, which changes the fantastic component of the SPP mode’s refractive index and shifts the frequency coinciding to the SPR peak. The change in resonant wavelength with temperature may be used to gauge this sensor’s sensitivity, and Equation (7) is used to describe the refracted index of it [54].

(7) $$S_{\lambda }=\frac{\Delta \lambda_{peak}}{\Delta T}\left(pm∕\mathrm{{}^{\circ}C}\right)\;$$

where $\Delta \lambda_{peak}$ is the range of the resonant wavelength shift and ΔT is the trade of temperature.

In Figure 9b, five temperature sensitivities are calculated: 3757 pm/°C, 3271 pm/°C, 2785 pm/°C, 2299 pm/°C, and 1813 pm/°C, respectively. The highest temperature sensitivity is 3757 pm/°C, when the temperature is close to 5 °C. This sensitivity has excellent results in SPR sensors with a diameter of about 20 μm and has a strong potential for development [55,56].

### 5.2. Refractive Index Sensing Performance

**Figure 10:**
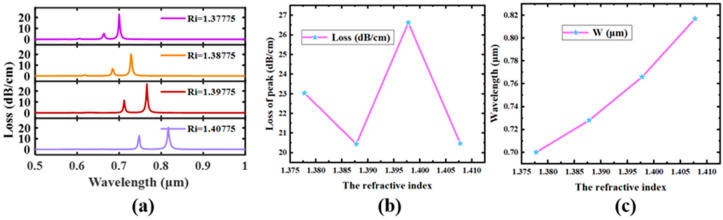
R_5_ = 1 μm, D = 30 nm, aa = 4 μm. (**a**) Effect of refractive index change on the loss peak. (**b**) The change in the peak value of the low-order SPP mode. (**c**) The change in wavelength where the maximum value of the low-order SPP mode is located.

In this paper, the RIS of the improved D-type dual-open-loop channel temperature sensor is analyzed. Figure 10 suggests the trade in the overall sensing performance and SPR height of the sensor at a refractive index of 1.37775–1.40775. As the set refractive index increases, the SPR height is red-shifted. The change in wavelength and its peak in the low-order SPP mode may be employed as an indicator of the sensor’s transparency since the exchange in the ERI of the SPP mode is directly caused by utilizing the change in the refractive index beneath the modulation of the segment matching point [57,58,59,60]. The sensor’s ability to detect brightness at certain refract indicia of the tested items is shown in Figure 10. In Figure 10c, four visible light sensitivities are calculated: 4847 nm/RIU, 3703 nm/RIU, 2559 nm/RIU, 1415 nm/RIU. As proven in Figure 10b, although the SPR loss peak is blue-shifted as the refractive index increases, the low-order SPP mode is the largest at the refractive index *Ri* = 1.39775.

### 5.3. Discussion

In conclusion, this paper presents a D-type double open-loop channel SPR-PCF for temperature sensing. The sensor has excessive temperature sensitivity and extremely good overall sensing performance in small diameter PCF sensors, which can extend the application range of PCF sensors and is of leap forward value. As shown in Table 2, the recently reported SPR sensors for temperature sensing are investigated in this paper [61,62,63]. Compared to them, it has high sensitivity as well as a simple nanostructure, and its small radius and simple nanostructure extends the application range of fiber optic temperature sensors, which is not less innovative.

## 6. Conclusions

We focus on the SPR-PCF sensor with D-shaped double open-loop channels nanostructure and examine its temperature sensitivity as well as RIS by finite element analysis. Compared with the conventional D-PCF, our proposed D-PCF has the following features. Firstly, we have two open-loop channels that facilitate the coupling of the Y-POL and SPP modes to enhance the sensitivity of the sensor while increasing its portability. A coating on the open ring of the ellipse can make production less complicated and minimize the production cost. The internal air holes in this SPR sensor are organized in a revolutionary periodic manner, with two binding holes beneath the Au film. This sketch promotes the energy leakage from the Y-POL quintessential mode into the plasma mannequin for coupling, but it also avoids the excessive outward growth of energy, which substantially improves the SPR effect. By optimizing three structural parameters, namely, the thickness of the Au film, the radius of the certain hole, and the elliptic semilength axis, the maximum temperature sensitivity of the low-order SPP mode is 3757 pm/°C and the maximum RIS is 4847 nm/RIU. Learning about this is important for lookup and development in the field of temperature sensing. The sensor is also suitable for the simultaneous detection of different substances with good portability. The SPR-PCF sensor proposed in this paper is not only anticipated to meet the wishes of temperature sensing applications, but also has the possibility of aiding in scientific diagnosis in the future. The fiber’s ultra-high sensitivity and unique shape underpin its first-rate potential for biomedical sensing applications, such as the detection of glucose, hemoglobin, and other biomolecules that use microfluidic channels. If different metal films are coated on the surfaces of the two grooves and filled with different analytes, the sensing of two physical parameters can be achieved simultaneously. An in-depth study can be conducted later.

## Figures and Tables

**Table 1:** Initial setting parameters.

Symbol	Parameter	Value
R_1_	radius of PCF	12 μm
R_2_	PDMS radius (assume it wraps the fiber to form a column)	13.5 μm
R_3_	No. 1 hole radius	1.5 μm
R_4_	No. 2 hole radius	1.2 μm
R_5_	No. 3 hole radius	1 μm
h	Width of polished surface (half side)	4√5 μm
d_1_	Distance from the center of the No. 2 hole to the hub of the fiber core	6 μm
d_2_	Distance between the hub of the No. 1 hole and the No. 2 hole	4 μm
d_1_ + d_2_	Distance from the hub of the No. 2 hole to the hub of the fiber core	10 μm
d_3_	The No. 3 hole from the vertical distance of the fiber core	4 μm
d_4_	The horizontal distance of the No. 3 hole from the fiber core	2 μm
D	Au film thickness	30 nm
aa	Length of the long axis of the groove	---------

**Table 2:** Performance evaluation of the proposed PCF-SPR temperature sensor mannequin.

Model	Refractive Index Sensitivity	Temperature Sensitivity
Model A 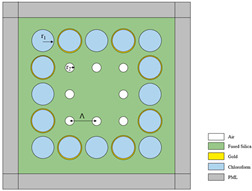 [61]	--------------	−10,400 pm/°C
Model B 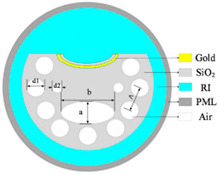 [62]	3330 nm/RIU	912 pm/°C
Model C 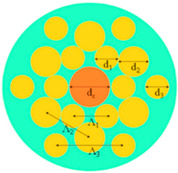 [63]	3550 nm/RIU	700 pm/°C
Model of this paper 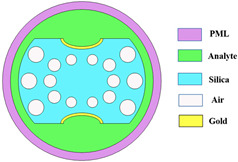	4847 nm/RIU	3757 pm/°C

## Data Availability

Publicly available datasets were analyzed in this study.
